# AKAP9 regulates activation-induced retention of T lymphocytes at sites of inflammation

**DOI:** 10.1038/ncomms10182

**Published:** 2015-12-18

**Authors:** Jan M. Herter, Nir Grabie, Xavier Cullere, Veronica Azcutia, Florencia Rosetti, Paul Bennett, Grit S. Herter-Sprie, Wassim Elyaman, Francis W. Luscinskas, Andrew H. Lichtman, Tanya N. Mayadas

**Affiliations:** 1 Center for Excellence in Vascular Biology, Department of Pathology, Brigham and Women's Hospital and Harvard Medical School, 77 Avenue Louis Pasteur, Boston, Massachusetts 02115, USA; 2 Department of Medical Oncology, Dana-Farber Cancer Institute and Harvard Medical School, 450 Brookline Avenue, Boston, Massachusetts 02115, USA; 3 Ann Romney Center for Neurologic Diseases, Brigham and Women's Hospital and Harvard Medical School, 77 Avenue Louis Pasteur, Boston, Massachusetts 02115, USA

## Abstract

The mechanisms driving T cell homing to lymph nodes and migration to tissue are well described but little is known about factors that affect T cell egress from tissues. Here, we generate mice with a T cell-specific deletion of the scaffold protein A kinase anchoring protein 9 (AKAP9) and use models of inflammatory disease to demonstrate that AKAP9 is dispensable for T cell priming and migration into tissues and lymph nodes, but is required for T cell retention in tissues. AKAP9 deficiency results in increased T cell egress to draining lymph nodes, which is associated with impaired T cell re-activation in tissues and protection from organ damage. AKAP9-deficient T cells exhibit reduced microtubule-dependent recycling of TCRs back to the cell surface and this affects antigen-dependent activation, primarily by non-classical antigen-presenting cells. Thus, AKAP9-dependent TCR trafficking drives efficient T cell re-activation and extends their retention at sites of inflammation with implications for disease pathogenesis.

## Abstract (web summary)

A-kinase anchoring protein 9 (AKAP9) is a scaffold protein that binds signalling proteins and regulates microtubules. Here the authors show that during inflammation AKAP9 in T cells is required for their reactivation and retention at the inflammation site and that its deletion protects from inflammation-induced organ damage.

## Graphical abstract

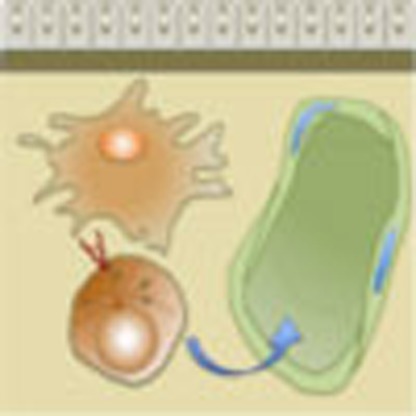

Maturation, differentiation and trafficking of T lymphocytes are critical for generating an effective immune response^1^,^2^. Dendritic cells (DCs) take up and process antigen at the site of inflammation and emigrate into secondary lymphoid organs, including lymph nodes. Circulating naïve T cells enter lymph nodes and differentiate and expand upon encountering their specific antigen loaded on major histocompatibility complex (MHC) class II molecules on DCs^3^. Mature effector T cells then leave lymphoid organs, enter the bloodstream, and migrate to sites of inflammation. There is mounting evidence that T cell recruitment to inflamed tissue occurs through a process that is largely antigen-independent^4^,^5^,^6^, whereas antigen recognition by tissue-resident antigen-presenting cells (APCs) results in T cell re-activation that elicits effector functions^7^,^8^. Effector T cells that fail to be activated *in situ* exit the inflamed tissue via afferent lymphatics and accumulate in the draining lymph node (dLN)^9^,^10^,^11^,^12^,^13^, guided by CCR7-CCL19/21 chemokine receptor/ligand cues^10^,^12^. However, intracellular molecular mechanisms that coordinate effector T cell retention versus egress remain largely unknown.

Several T cell functions including T cell homing and motility, conjugate formation with APCs, T cell antigen receptor (TCR) recycling and migration into inflamed tissues are coordinated by the actin and microtubule (MT) network^14^. MTs are dynamic structures that undergo growth and catastrophe, which are important for cell division, vesicular trafficking and migration^15^. The scaffold protein A kinase anchoring protein 9 (AKAP9, AKAP450), present in the Golgi and centrosome of most cells, is emerging as a regulator of MTs emanating from these MT organizing centres^15^,^16^,^17^, particularly the cis-Golgi^15^. AKAP9 has been implicated in processes that may rely on MTs such as the polarization and migration of T cells^18^ as well as the formation of the immune synapse with APCs via effects on a T cell integrin, LFA-1 (ref. 19) in human T cell lines. MTs from the Golgi represent a distinct MT subpopulation that does not rely on centrosomal nucleation and regulates specific cellular tasks, which are beginning to be elucidated^20^. Thus, AKAP9 may regulate a subset of MTs that impact defined cellular functions in T cells and other cell types. Indeed, the normal viability of AKAP9 global-deficient mice^21^ infer circumscribed rather than global roles for AKAP9 in MT functions.

To explore the physiological role of AKAP9 in T cell functions, we generated mice with a conditional deletion of AKAP9 specifically in CD4 and CD8 T cells using Cre-driven by the CD4 promoter^22^, which we refer to as AKAP9^cko/CD4^. We show that AKAP9 deficiency did not impair T cell priming, expansion or migration into tissues. Rather, it prevented re-activation and retention of T cells in inflamed tissue in two clinically relevant disease models, anti-glomerular basement membrane (GBM) nephritis and experimental autoimmune encephalitis (EAE), a model of multiple sclerosis. The impaired retention in AKAP9^cko/CD4^ mice correlated with protection from developing organ damage. *In vitro*, AKAP9 was required for optimal MT-dependent endosomal TCR recycling to the surface of activated T cells. The functional consequences of this was apparent only under suboptimal antigen-presentation conditions as is observed with B cells or tissue-resident APCs. Together, our studies uncovered an important role for AKAP9 in TCR re-activation and T cell retention in peripheral tissue, which has consequences for tissue damage in inflammatory diseases.

## Results

### AKAP9 is not involved in T cell recruitment or priming

**Figure 1:**
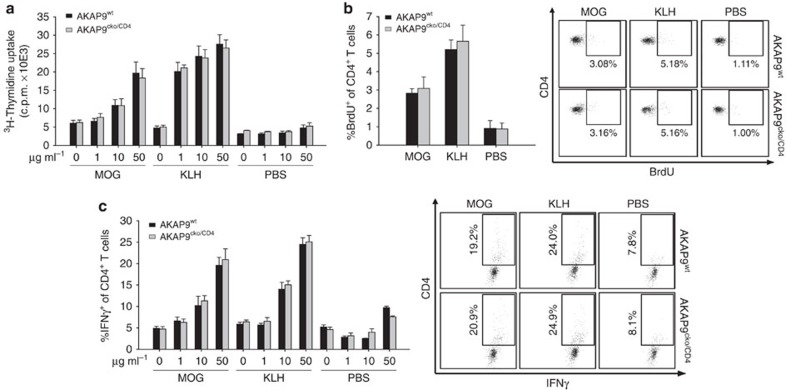
Priming of CD4^+^ T cells is unaffected in AKAP9^cko/CD4^ mice. (**a**) Proliferation of T cells in lymph node suspensions recovered 4 days after foot pad immunization with MOG, keyhole limpet hemocyanin (KLH) or PBS from draining inguinal lymph nodes and co-incubated with increasing concentrations of the immunizing peptide (cells from PBS immunized mice were incubated with MOG peptide). Data are presented as mean uptake of ^3^H-Thymidine ±s.e.m., *n*=5. (**b**) Mean incorporation of BrdU over 24 h in CD4+ T cells in draining lymph nodes on day 4 after immunization with either MOG, KLH or PBS ±s.e.m., *n*=5. Exemplary dot plots of BrdU staining are provided, gated versus CD4. (**c**) Mean frequency of IFNγ-positive cells in draining lymph nodes on day 4 after immunization with either MOG, KLH or PBS following incubation with increasing concentrations of the immunizing peptide (cells from PBS immunized mice were incubated with MOG peptide) after 48 h ±s.e.m., *n*=5. Exemplary dot plots of IFNγ staining of samples incubated with 50 μg ml^−1^ are provided, gated versus CD4.

Mice with conditional deletion of AKAP9 in T cells (AKAP9^cko/CD4^) were generated on a C57Bl/6 strain and confirmed to lack AKAP9 protein in CD4^+^ T cells (Supplementary Fig. 1). The mice were viable and had no detectable defects under specific-pathogen-free housing conditions. Wild-type control (AKAP9^wt^) and AKAP9^cko/CD4^ mice had comparable spleen size, T cell numbers in the spleen, lymph nodes and blood, and a similar distribution of effector/memory markers CD44, CD62L, as well as the TCR and co-stimulatory molecules (Supplementary Fig. 2a–d). Interestingly, AKAP9^wt^ T cells differentiated to T_H_1 have increased AKAP9 message compared with naïve cells as assessed by quantitative reverse transcription–PCR (5.57±0.16-fold). AKAP9 has been implicated in functions of LFA-1 (refs 18, 19), an integrin necessary for T cell trafficking to non-lymphoid and lymphoid tissue^1^,^2^ and APC/ T cell conjugate formation^23^ with LFA-1 deficiency leading to defects in T cell responses including antigen-dependent activation^24^. However, we observed no alterations in the recruitment of effector T cells in the tumour-necrosis factor (TNF)-induced air pouch model (Supplementary Fig. 3a), a LFA-1-dependent model of lymphocyte recruitment^25^, in the homing of transferred, naïve T cells to peripheral lymph nodes (Supplementary Fig. 3b) or in LFA-1-dependent cell adhesion, chemotaxis and transmigration *in vitro* (Supplementary Fig. 3c–f). Consistent with these findings, T cell priming was intact in AKAP9^cko/CD4^ mice following immunization with keyhole limpet hemocyanin or myelin oligodendrocyte glycoprotein (MOG) peptide (Fig. 1a–c).

### T_H_1 effector activation in glomerulonephritis requires AKAP9

To examine the role of AKAP9 in T effector cell functions *in vivo*, we used a model of crescentic glomerulonephritis (GN) where mice were pre-immunized with CFA/IgG and then administered an antibody against the GBM. This provokes a T_H_1-predominant nephritis^26^,^27^,^28^ to an autoantigen that remains to be defined and may be released from kidney tissue upon neutrophil^29^, monocyte^30^ or CD8-mediated destruction of cells targeted by the antibody^31^.

**Figure 2:**
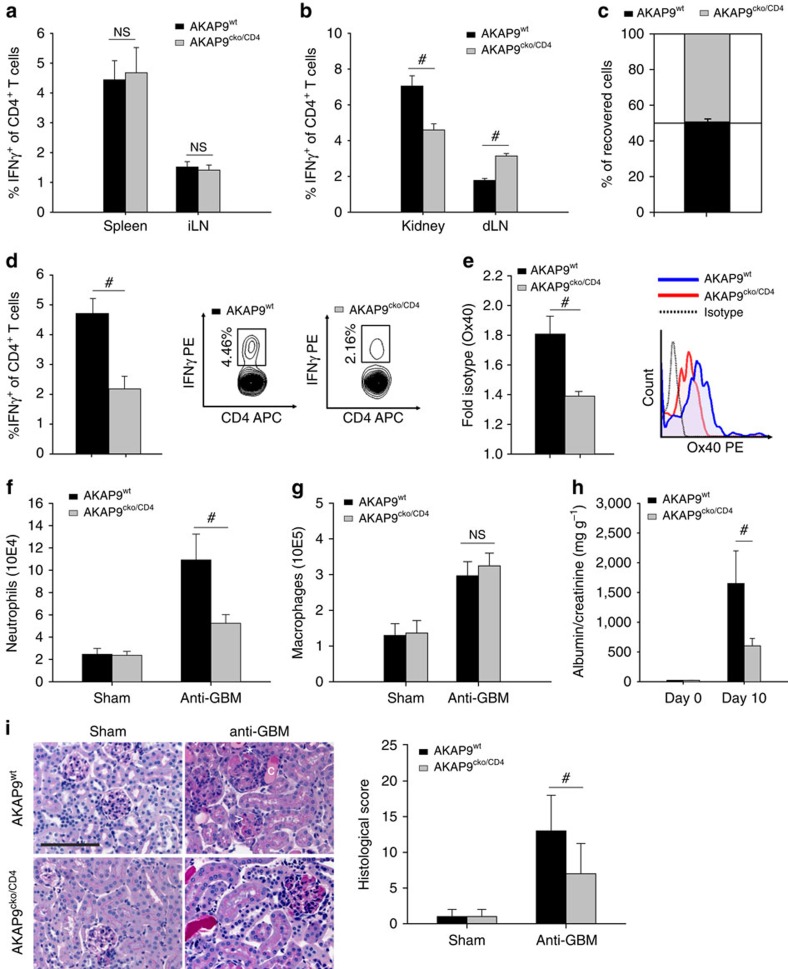
T_H_1 effector activation in glomerulonephritis requires AKAP9. (**a**) Frequency of IFNγ-positive cells, following *in vitro* PMA/ionomycin stimulation, in CD4^+^ T cell isolates from the spleen and immunizing lymph node of AKAP9^cko/CD4^ and AKAP9^wt^ mice 10 days following induction of anti-GBM nephritis. Data presented as mean frequency of cells ±s.e.m., *n*=7. (**b**) As in **a** for the kidney and draining lymph node. (**c**) T_H_1 cells from AKAP9^cko/CD4^ and AKAP9^wt^ mice were labelled and co-transferred into wild-type animals on day 10 following induction of anti-GBM nephritis. Accumulation was analysed in the kidney 3 h after adoptive transfer. Data are mean percentage of recovered cells ±s.e.m., *n*=5. (**d**) As in **a** for CD4^+^ T cells recovered from the kidney but without PMA/ionomycin treatment before intracellular cytokine staining. Plots are representative FACS panels of IFNγ expression in AKAP9^cko/CD4^ and AKAP9^wt^ control cells. (**e**) Ox40 expression of kidney CD4^+^ T cells of **a**, presented as fold isotype staining ±s.e.m. Histogram is a representative of Ox40 staining (*x* axis log scale). (**f**) Neutrophil (CD45^+^Ly6G^+^Ly6B.2^+^) and (**g**) Macrophage (CD45^+^CD11b^+^F4/80^+^) infiltration in kidneys of **a**, presented as number of cells ±s.e.m. (**h**) Mean albuminuria at day 0 and 10 after induction of anti-GBM nephritis of (**a**), presented as mg urinary albumin per g of urinary creatinine ±s.e.m. (**i**) Left panel: Representative micrographs of PAS staining of kidney sections. Asterisk indicates crescent formation, ‘c' indicates tubular casts and ‘>' indicates glomerular thrombosis. Scale bar, 100 μm. Right panel: Median histological scoring based on hypercellularity, glomerular thrombosis, crescent formation, tubular casts and morphologic glomerular irregularity±interquartile range. #*P*<0.05. NS, not significant.

Crescentic GN was induced in AKAP9^wt^ and AKAP9^cko/CD4^ mice and animals of both groups had similar swelling at the site of the sensitizing IgG/adjuvant delayed-type hypersensitivity reaction in the footpad (4.80±0.34 mm in AKAP9^wt^ versus 4.84±0.30 mm in AKAP9^cko/CD4^). T_H_1 effector cells in the spleen and immunizing lymph node, as assessed by the fraction of interferon (IFN)γ-positive T cells, were present in equal numbers (Fig. 2a), indicating comparable generation of effector cells and thus T cell priming. In the kidney, total T cell accumulation 10 days post injection of the anti-GBM serum was comparable in wild-type and AKAP9^cko/CD4^ mice (AKAP9^wt^ 9.7±0.7 × 10^4^ versus AKAP9^cko/CD4^ 9.1±0.6 × 10^4^ cells). However, T_H_1 effector T cells in the kidney were reduced in AKAP9^cko/CD4^ animals, with a concomitant accumulation in the dLNs (Fig. 2b). Similar results were obtained for T_H_17 cells, albeit they were much less abundant (AKAP9^wt^ 1.93%±0.33% versus AKAP9^cko/CD4^ 1.37%±0.19% in kidney, 1.24%±0.13% versus 1.87%±0.35% in dLNs). To formally determine whether defects in recruitment may account for these phenotypes, differentially labelled *in vitro* differentiated AKAP9^wt^ and AKAP9^cko/CD4^ T_H_1 cells were adoptively co-transferred via tail vein injection at day 10 after induction of glomerulonephritis. We observed equal accumulation of AKAP9^cko/CD4^ and AKAP9^wt^ cells (Fig. 2c), confirming intact recruitment of cells in the absence of AKAP9. No T cell accumulation of either genotype was observed in the dLNs at this early time point. Differences in apoptosis did not account for the reduced number of effector cells in the kidney as the amount of Annexin V^+^ CD4^+^ T cells, albeit low, were similar in AKAP9^wt^ and AKAP9^cko/CD4^ animals (AKAP9^wt^ 2.63±0.25% versus AKAP9^cko/CD4^ 2.41±0.18%). The observed reduction in renal T_H_1 effector cells in the absence of recruitment defects, and the associated increase in their accumulation in the dLN suggest impaired retention or increased egress of T_H_1 cells from the nephritic kidney of AKAP9^cko/CD4^ mice.

Local presentation of antigen by intrinsic renal cells expressing MHC class II plays a key role in T cell-mediated renal injury in crescentic GN^32^, and retention of T cells in other disease models depends upon their sufficient activation at the site of inflammation^10^,^12^. In contrast to primary T cell activation, tools to assess T cell re-activation are limited. Previous studies have used intracellular staining of effector cytokines without additional *in vitro* stimulation for this purpose^33^,^34^. Using this approach, we found a smaller fraction of CD4+ T cells recovered from the tissue of AKAP9^cko/CD4^ mice that stained positive for the effector cytokine IFNγ (Fig. 2d), implying that renal accumulated AKAP9^cko/CD4^ T cells were less efficiently re-stimulated *in situ*. To confirm these results, we evaluated the expression of the co-stimulatory molecule Ox40 on CD4^+^ T cells recovered from the kidney, as expression of this surface molecule has been reported to be indicative of tissue re-stimulation^33^,^34^. AKAP9^cko/CD4^ T cells had significantly lower expression of Ox40 compared with WT controls (Fig. 2e). Together, these data suggest that AKAP9-deficient T cells were less efficiently re-stimulated in the inflamed kidney compared with their WT counterparts.

The relative deficiency of both the total and re-stimulated number of T_H_1 cells in the kidney of AKAP9^cko/CD4^ mice was associated with a decrease in infiltrating neutrophils (Fig. 2f) but not macrophages (Fig. 2g) following crescentic GN. The differential effects on neutrophils versus macrophages are not clear but may be linked to an overall reduction in T_H_17 or T_H_1 cytokines that potentially regulate neutrophil accumulation^35^. Overall, AKAP9^cko/CD4^ animals had reduced glomerular injury as demonstrated by decreased albuminuria (Fig. 2h) and renal histopathological changes (Fig. 2i) compared with wild-type controls.

### Impaired retention associates with reduced TCR signal strength

**Figure 3:**
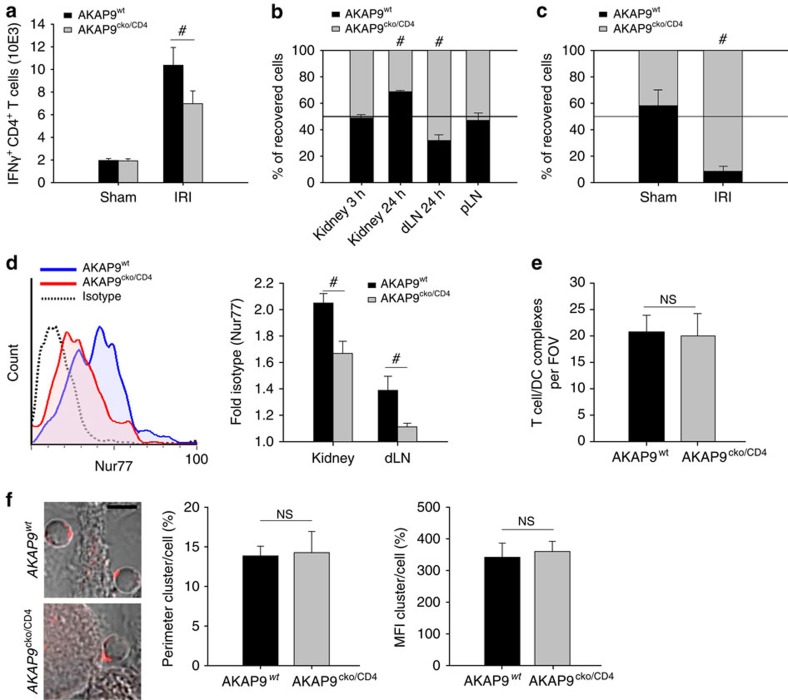
Impaired retention associates with reduced TCR signal strength. (**a**) CD4^+^ T cell infiltration in kidneys of AKAP9^cko/CD4^ and AKAP9^wt^ mice following sham surgery or 24 h after ischaemia-reperfusion injury. Data presented as mean number of IFNγ-positive cells ±s.e.m. recovered from the kidney and analysed in the presence of PMA/ionomycin, *n*=5. (**b**) T_H_1 cells from AKAP9^cko/CD4^ and AKAP9^wt^ mice were differentially labelled and co-transferred into wild-type animals subjected to ischaemia followed by reperfusion. Accumulation was analysed at the indicated times after reperfusion in the kidney and in the draining and peripheral lymph nodes. Data are mean percentage of recovered cells ±s.e.m., *n*=6. (**c**) T_H_1 cells of AKAP9^cko/CD4^ and AKAP9^wt^ mice were introduced via subcapsular injection into wild-type mice 12 h after reperfusion and draining lymph nodes were analysed 24 h after to enumerate the recovered % of each population, *n*=6. (**d**) Nur77 expression in transferred Vβ8^+^ T_H_1 cells recovered from SEB-pulsed kidneys or draining lymph nodes 24 h after reperfusion injury. Left panel: Representative histogram of Nur77 staining (*x* axis linear scale) in AKAP9^cko/CD4^ and AKAP9^wt^ T_H_1 cells from the kidney. Right panel: Quantification presented as fold isotype staining ±s.e.m., *n*=4. (**e**) Conjugate formation *in situ* of labelled T_H_1 cells co-incubated with kidney slices from CX_3_CR1-GFP mice pulsed with SEB in the subcapsular space. Cell–cell interactions were quantified as contacts between two flattened but not round cells. Data presented as mean number of contacts per field of view (FOV) ±s.e.m., *n*=3. (**f**) Confocal microscopy of T cells sorted for TCR Vβ8.1/8.2, co-incubated with SEB-pulsed bmDCs and stained for LFA-1. Scale bar, 10 μm. Clustering was quantified as the percentage of the cell perimeter displaying the cluster over the total cell perimeter as well as the percentage of the mean mean fluorescence intensity (MFI) within the cluster over the mean MFI of the total cell membrane. Presented are means ±s.e.m., *n*=4, >15 cells per *n*. #*P*<0.05. NS, not significant.

To confirm our key findings of intact recruitment but reduced retention of T_H_1 effector cells in the nephritic kidney of AKAP9^cko/CD4^ mice and further interrogate the effects of AKAP9 deficiency on T cell re-stimulation *in situ,* we exploited a model of renal ischaemia-reperfusion wherein recruitment of IFNγ-producing CD4^+^ T_H_1 cells occurs as early as 3 h after reperfusion injury^36^,^37^. Thus, this model has the advantage of being temporally defined and amenable to evaluating events immediately proximal to the injurious stimulus. AKAP9^cko/CD4^ and AKAP9^wt^ mice were subjected to 32 min ischaemia and analysed 24 h after reperfusion. As in crescentic glomerulonephritis, the number of renal T_H_1 cells was reduced in AKAP9^cko/CD4^ mice compared with wild-type counterparts (Fig. 3a). Differentially labelled AKAP9^wt^ and AKAP9^cko/CD4^ T cells adoptively transferred after induction of reperfusion injury similarly accumulated in the post-ischemic kidney 3 h after reperfusion (Fig. 3b), whereas none were recovered in the dLNs at this time point, indicating that recruitment of effector cells is not AKAP9 dependent. After 24 h, a significant reduction in the number of adoptively transferred AKAP9^cko/CD4^ T cells in the kidney was observed (Fig. 3b) that was accompanied by a reciprocal increase of the AKAP9^cko/CD4^/AKAP9^wt^ ratio in the dLNs (Fig. 3b). No significant proliferation of endogenous renal T cells was observed at 24 h following reperfusion in either AKAP9^wt^ or AKAP9^cko/CD4^ animals as assessed by 5-bromodeoxyuridine (BrdU) incorporation in CD4^+^ cells (AKAP9^wt^ 0.63±0.21% versus AKAP9^cko/CD4^ 0.45±0.14%), which indicates that defects in proliferation do not account for the observed reduction of renal T cells in AKAP9-deficient animals. To directly assess egress of T cells from the renal tissue, we co-transferred labelled AKAP9^wt^ and AKAP9^cko/CD4^ T_H_1 cells directly into the subcapsular space of the kidney of wild-type animals 12 h after reperfusion and assessed accumulation in the dLNs. Notably, the recovered cells from the lymph nodes were almost exclusively AKAP9^cko/CD4^ T cells (Fig. 3c), indicating impaired retention in the kidney.

To examine TCR activation *in situ*, we designed a superantigen-dependent approach. Carboxyfluorescein succinimidyl ester (CFSE)-labelled AKAP9^wt^ or AKAP9^cko/CD4^ T_H_1 cells were transferred into wild-type animals following ischemia-reperfusion and subcapsular injection of the superantigen streptococcal enterotoxin-B (SEB) to provide local antigen for Vβ8.1/8.2^+^ T cells, a technique comparable to local antigen delivery reported previously^4^. After 24 h, Vβ8.1/8.2^+^CFSE^+^ T cells in the kidney and dLNs were evaluated for endogenous Nur77, the levels of which correlate with TCR signal strength^38^. Transferred AKAP9^wt^ T cells had significantly higher Nur77 expression in the kidney versus the dLN (Fig. 3d). Notably, AKAP9^cko/CD4^ cells had lower Nur77 expression in the kidney and even less in the dLN versus the wild-type counterparts (Fig. 3e), indicating weaker TCR signals in these cells in both these compartments. Impaired activation of AKAP9-deficient T cells in the tissue was not the result of impaired conjugate formation as viable organ slices of the kidney from CX_3_CR1-GFP mice^4^, when pulsed with SEB, equally supported interactions with AKAP9^wt^ and AKAP9^cko/CD4^ T cells (Fig. 3e), and LFA-1 clustering at T cell/APC contacts in conjugates was also unaffected by AKAP9 deficiency *in vitro* (Fig. 3f). Together, the data suggest that the cause of the reduced TCR signalling strength in AKAP9^cko/CD4^ T cells is downstream of conjugate formation.

### T-cell retention correlates with the development of EAE

**Figure 4:**
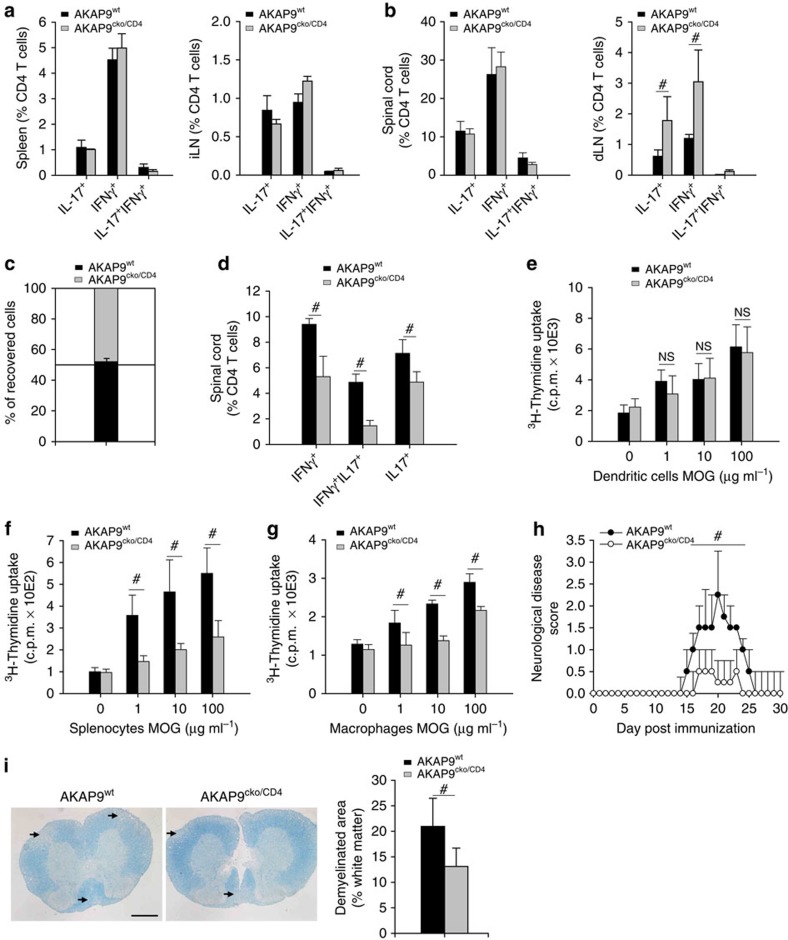
T-cell retention correlates with the development of EAE. (**a**) Intracellular cytokine analysis following PMA/ionomycin treatment of CD4^+^ T cells recovered from the spleen (left panel) and immunizing lymph node (right panel) 10 days after immunization. Data presented as mean percentage of CD4^+^ T cells ±s.e.m., *n*= 5. (**b**) Intracellular cytokine analysis, after *in vitro* PMA/ionomycin treatment, of CD4^+^ T cells from the spinal cord (left panel) and draining LN (right panel) at day 15 as in **a**. (**c**) T_H_1 cells from AKAP9^cko/CD4^ and AKAP9^wt^ mice were differentially labelled and co-transferred into wild-type animals on day 15 following induction of EAE. Accumulation was analysed in the spinal cord 3 h after adoptive transfer. Data are mean percentage of recovered cells ±s.e.m., *n*=5. (**d**) Intracellular cytokine analysis, without PMA/ionomycin, of CD4^+^ T cells harvested from spinal cords in **b** presented as mean percentage of CD4^+^ T cells ±s.e.m. (**e**) Proliferation of T cells 10 days after immunization co-incubated with dendritic cells and increasing concentrations of the immunizing peptide, presented as mean uptake of ^3^H-Thymidine ±s.e.m., APC/T-cell ratio 1:2, *n*=5. (**f**) Proliferation as in **e** with mitomycin-treated splenocytes as APCs, APC/T-cell ratio 5:1, *n*=4. (**g**) Proliferation as in **e** with bone marrow-derived macrophages, APC/T-cell ratio, 1:2, *n*=5. (**h**) Median disease score of AKAP9^cko/CD4^ and AKAP9^wt^ mice over time ±IQR (interquartile range) *n*=8. (**i**) Left panel: Representative micrographs of Luxol blue-stained spinal cords of AKAP9^cko/CD4^ and AKAP9^wt^ mice at day 30 post immunization. Arrows point to areas of demyelination. Scale bar, 100 μm. Right panel: Quantification of demyelinated white matter. #*P*<0.05. NS, not significant.

To determine whether our findings extend to other organs and are recapitulated for T cell responses to a defined antigen, we evaluated T cell behaviour and disease outcomes following EAE, induced by immunization of mice with MOG peptide. The immunological response to MOG, a key determinant of disease^39^, was similar in AKAP9^wt^ and AKAP9^cko/CD4^ animals (Fig. 1). In line with these findings, differentiation and expansion of T cell populations in the spleen and immunizing lymph nodes (axillary, Fig. 4a) 10 days after peptide immunization were similar between the groups. In spinal cords, a mild, non-significant reduction in AKAP9^cko/CD4^ CD4^+^ T cells was detected compared with AKAP9^wt^ samples (AKAP9^wt^ 2.2±0.1 × 10^4^ versus AKAP9^cko/CD4^ 1.8±0.2 × 10^4^) at the peak of the disease. Notably, although comparable numbers of T_H_1 and T_H_17 cell were present in the spinal cord as evaluated by cells producing IFNγ and interleukin (IL)-17, respectively (Fig. 4b), an increase in accumulation of both T_H_1 and T_H_17 cells was observed in the dLNs of AKAP9^cko/CD4^ mice compared with wild-type controls (Fig. 4b). Proliferation is unlikely to contribute to observed differences, as BrdU uptake of effector cells in the spinal cord is negligible^4^. Moreover, adoptive transfer of *in vitro* differentiated effector cells showed similar recruitment of AKAP9^wt^ and AKAP9^cko/CD4^ T cells to the spinal cord (Fig. 4c) and analysis of Annexin V^+^ on CD4+ cells recovered from the spinal cords revealed similar levels of apoptosis in both groups (AKAP9^wt^ 5.53±0.32% versus AKAP9^cko/CD4^ 5.98±0.49%). To evaluate T cell re-activation *in vivo*, we conducted intracellular cytokine analysis of CD4^+^ cells isolated from spinal cords of animals in the absence of *in vitro* phorbol myristate acetate (PMA)/ionomycin, as PMA/ionomycin treatment stimulates cytokine production in these cells and is thus primarily used to enumerate effector cell populations. Substantially fewer IL-17- and IFNγ-producing cells were present in AKAP9^cko/CD4^ versus AKAP9^wt^ animals when analysed in the absence of *in vitro* PMA/ionomycin treatment (Fig. 4d).

To explore if antigen-dependent re-stimulation of AKAP9-deficient T cells was impaired *in vivo*, we purified CD4^+^ T cells from spleens of MOG-immunized mice and performed co-culture experiments with MOG-pulsed DCs. We observed no difference in thymidine uptake under these conditions (Fig. 4e). In contrast, when co-incubated with MOG-pulsed splenocytes or bone marrow-derived macrophages, AKAP9^cko/CD4^ cells revealed severely impaired thymidine uptake, even at high MOG concentrations (Fig. 4f,g). These data suggest that AKAP9^cko/CD4^ T cells have impaired activation under suboptimal antigen presentation by tissue macrophages, but that this may be overcome by more efficient antigen presentation and co-stimulatory signalling delivered by mature splenic DCs. Importantly, antigen-presentation in non-lymphoid tissue has been reported to be less efficient compared with DC-mediated T cell maturation in lymphoid organs^32^,^40^,^41^,^42^.

AKAP9 deficiency and associated defects in T cell activation in tissues were associated with a reduction of disease in EAE as determined by neurological disease scoring (Fig. 4h) and histological evaluation (Fig. 4i). Disease scores in the relatively disease-resistant C57BL/6 wild-type animals were mild, but comparable to scores previously described in the B6 strain background^43^,^44^.

### AKAP9 is required for re-stimulation by non-classical APCs

**Figure 5:**
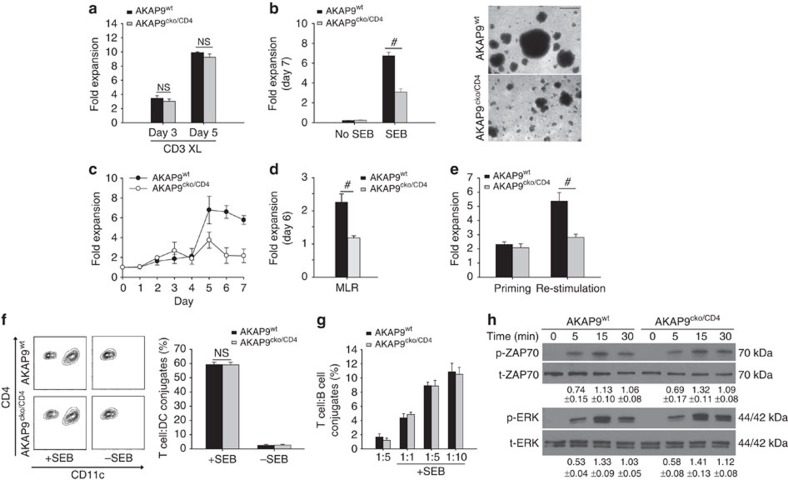
AKAP9 is required for restimulation by non-classical APCs. (**a**) Proliferation under T_H_1-differentiating conditions of naïve AKAP9^wt^ and AKAP9^cko/CD4^ CD4^+^ T cells after 3 days of TCR/CD3ɛ-crosslinking and an additional 2 days of incubation, presented as mean fold expansion (cell number/cell number at day 0) ±s.e.m., *n*=5. (**b**) Left panel: Proliferation following 7 days of co-incubation with splenocytes in conditions as in **a** in the absence or presence of SEB, *n*=5. Right panel: Representative micrographs of T cell clusters on day 5 of culture. Scale bar, 100 μm. (**c**) T cell proliferation with SEB over time, *n*=3. (**d**) Mixed lymphocyte reaction: Proliferation after 6 days of incubation with allogeneic Balb/c splenocytes, *n*=4. (**e**) Re-stimulation reaction: Naïve AKAP9^wt^ and AKAP9^cko/CD4^ CD4^+^ T cells were incubated with SEB-loaded bmDCs for 5 days, rested for 3 days (Priming) and then re-stimulated with SEB-loaded splenocytes (Re-stimulation). ±s.e.m., *n*=5. (**f**) Conjugate formation with SEB-pulsed DCs. Left panel: Representative FACS plots of CD11c versus CD4 (pre-gated for TCR Vβ8.1/8.2). Right panel: Mean percentage of CD11c-positive CD4^+^TCR Vβ8.1/8.2^+^ events ±s.e.m., *n*=4. (**g**) Conjugate formation with SEB-pulsed splenocytes at indicated T cell:splenocyte ratios, presented as mean percentage of CD19^+^ events of CD4^+^ T cells ±s.e.m., *n*=3. (**h**) Phosphorylation of ZAP70 and ERK in AKAP9^wt^ and AKAP9^cko/CD4^ T cells after co-incubation with SEB-pulsed splenocytes for the indicated times in minutes. Quantification is the relative density of phospho-specific signals to corresponding total protein signals ±s.e.m., *n*=3. #*P*<0.05. NS, not significant.

To understand why AKAP9-deficient CD4^+^ T cells fail to effectively re-stimulate *in vivo*, we turned to *in vitro* culture experiments. Splenic CD4^+^ T cells from naïve AKAP9^cko/CD4^ and wild-type controls showed similar proliferation under T_H_1 differentiating conditions following CD3ɛ-crosslinking (Fig. 5a) and differentiated with similar efficiency under T_H_1 (AKAP9^wt^ 64.7±3.1% versus AKAP9^cko/CD4^ 63.9±4.7%, *n*=5) and T_H_2 conditions (AKAP9^wt^ 13.2±1.2% versus AKAP9^cko/CD4^ 14.8±2.1%, *n*=4). Moreover, similar proliferation was observed using bone marrow-derived dendritic cells (bmDCs) as APC in response to the superantigen SEB (see Fig. 5e, ‘Priming'), which links class II MHC of APCs to Vβ8.1 or 8.2 TCRs and models MHC-dependent activation of T cells^45^. In contrast, a marked proliferation defect in these cells was observed in response to SEB presented by splenocytes (Fig. 5b,c), which is consistent with results from MOG-challenged animals (Fig. 4g-i). A similar proliferative defect in AKAP9 deleted T cells was observed in a mixed lymphocyte reaction with BALB/c splenocytes as APCs (Fig. 5d), a reaction in which T cells from H-2^b^ mice are activated because of allo-reactivity to H-2^d^-expressing APCs^46^. Together, these data demonstrate that AKAP9 is required for T cell proliferation and/or survival following TCR engagement only under suboptimal antigen presentation characteristic of non-classical APCs. Next, we directly evaluated T cell priming and re-activation in the same population of T cells. Naïve CD4^+^ T cells harvested from AKAP9^wt^ and AKAP9^cko/CD4^ mice were primed with SEB loaded bmDCs under T_H_1 differentiating conditions for 5 days, given 3 days of rest in IL-2 containing media followed by re-stimulation with SEB-loaded splenocytes for 3 days. T cell expansion during primary priming with bmDCs was similar in both groups (Fig. 5e, ‘Priming'). However, upon re-stimulation, AKAP9^cko/CD4^ T cells exhibited a greatly reduced proliferative response compared with AKAP9^wt^ cells (Fig. 5e, ‘Re-stimulation'). Notably, the difference in proliferation between Fig. 5b,c (splenocyte/naïve T cells under Th1 differentiating conditions) and Fig. 4e (bmDCs/naïve T cells) is that the inflammatory cytokines added in the former prompt a more productive and stable proliferative response. These results corroborate our *in vivo* data, which showed that AKAP9 is dispensable for T cell priming in lymph nodes, but is required for the effective re-stimulation of primed T cells at sites of inflammation by tissue resident APCs.

Defective re-stimulation is not a consequence of impaired conjugate formation with APCs as comparable number of Vβ8.1/Vβ8.2^+^CD4^+^ AKAP9^wt^ and AKAP9^cko/CD4^ T cells bound to bmDCs (CD11c^+^, Fig. 5f) and splenocytes (CD19^+^, B cells, Fig. 5g) could be identified following co-incubation. There was also no difference in the rapid ZAP70 or ERK phosphorylation in T cells after co-incubation with SEB-pulsed splenocytes (Fig. 5h), suggesting that signalling events upon initial TCR activation are unaffected by AKAP9 deficiency.

### Impaired TCR recycling in AKAP9-deficient T cells

**Figure 6:**
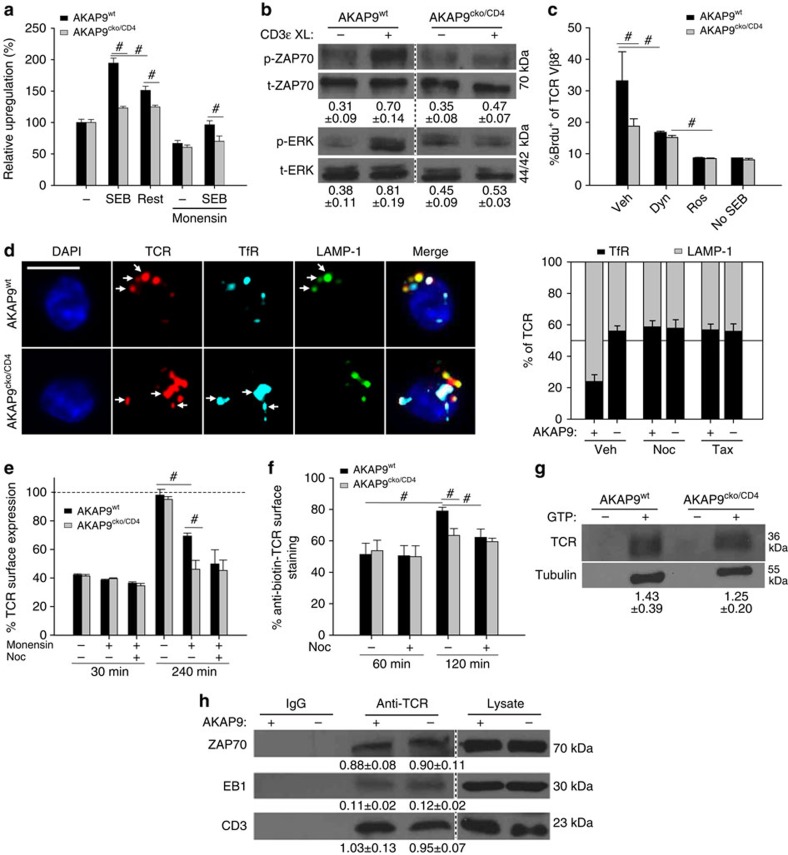
Impaired TCR recycling in AKAP9-deficient T cells. (**a**) Upregulation of TCR-β after incubation with or without SEB-loaded bmDCs as mean percent staining over baseline expression untreated cells with or without addition of Monensin, and after 4 h of rest ±s.e.m., *n*=4. (**b**) Representative western blot of ZAP70 and ERK phosphorylation after overnight incubation of T cells with SEB-loaded bmDCs with or without CD3ɛ-crosslinking (XL). Quantification is relative density of phospho-specific signals to corresponding total protein signals ±s.e.m., *n*=3. (**c**) Mean BrdU uptake ±s.e.m. of cells co-incubated with SEB-loaded splenocytes with or without the clathrin inhibitor Dynasore (Dyn), the cyclin-dependent kinase 1 inhibitor Roscovitine (Ros) or with no SEB, *n*=3. (**d**) T cells (+, AKAP9^wt^; −, AKAP9^cko/CD4^) were activated using plate-bound anti-CD3ɛ and analysed 30 min after replating on fresh plates. Co-localization of TCR with an early (TfR) and late (LAMP-1) endosome marker after re-normalization to focus on endosomes. Treatment with nocodazole (Noc), Taxol (Tax) or vehicle (Veh) was done before incubation with plate-bound anti-CD3ɛ. Scale bar, 10 μm. Data presented as mean percentage of distribution to either compartment ±s.e.m. Representative confocal micrographs of TCR-β, TfR and LAMP-1. Arrows in AKAP9^wt^ illustrate co-localization of TCR with LAMP-1. Arrows in AKAP9^cko/CD4^ illustrate co-localizaton of TCR with TfR. Data are presented as percent overlap with TCR±s.e.m., *n*=3. (**e**) Re-surfacing of TCR surface expression after CD3ɛ-crosslinking. T cells were incubated with plate-bound anti-CD3ɛ, replated for 30 min or 240 min and assessed by FACS. All cells were treated with cycloheximide and, in some cases, also with monensin and/or Noc as indicated. Data are presented as mean percent compared with untreated cells (dashed line) ±s.e.m., *n*=4. (**f**) Recycling of endocytosed TCR. Cells were stained with biotin anti-TCR after plate-bound anti-CD3ɛ crosslinking and replating for 2 h to allow recycling before staining with streptavidin-PE. Presented as mean percent of staining intensity without incubation ±s.e.m., *n*=3. (**g**) Western blot of microtubule co-sedimentation assay. Quantification is the relative density of TCR-β to corresponding α-tubulin signal ±s.e.m., *n*=4. (**h**) Western blot of co-immunoprecipitations with anti-TCR antibody or IgG isotype control. Co-immunoprecipitates were blotted for EB1, ZAP70 or CD3. ZAP70 and CD3 served as controls. Quantification is the mean ratio of co-immunoprecipitated signal over lysate ±s.e.m., *n*=4. #*P*<0.05.

As both proximal (ZAP70) and distal (ERK) TCR signalling events were found to be intact in AKAP9-deficient cells, we hypothesized that differences in intracellular trafficking of the TCR account for the differences in re-stimulation observed *in vivo*. Previous studies have reported that CD4^+^ T cells upregulate TCR molecules on the surface after antigen-dependent re-stimulation^47^, and that even minor changes in TCR surface concentrations can significantly impact the threshold of T cell activation and subsequent cytokine generation following antigen-dependent stimulation^48^. AKAP9^wt^ and AKAP9^cko/CD4^ CD4^+^ T cells had comparable levels of TCR molecules under baseline conditions (Supplementary Fig. 2d). However, after overnight incubation with SEB-loaded DCs, the increase in surface expression of TCRβ observed in AKAP9^wt^ CD4^+^ T cells (Fig. 6a) was significantly blunted in AKAP9^cko/CD4^ cells. Similar results were obtained for CD3ɛ (Supplementary Fig. 2e). This difference in upregulation was also observed in T cells treated with the ionophore monensin, which inhibits transport from the Golgi and lysosomal degradation^49^, thus leaving the recycling of early endosomes as the sole source of TCR surface replenishment (Fig. 6a). This suggested that TCR endosomal trafficking is AKAP9 dependent. Interestingly, after an additional 4 h of incubation without APCs, TCR surface expression declined (Fig. 6a ‘rest'), indicating that the observed upregulation is transient. Thus, AKAP9 may be essential for T cell activation only in settings of serial APC contacts, as reported in non-lymphoid tissue^7^,^8^. As defects in receptor surface display impact signalling^48^, we analysed CD3ɛ-crosslinking induced signalling in cells pre-stimulated with SEB-loaded bmDCs and observed significantly less activation of the proximal kinase ZAP70 and the downstream kinase ERK in AKAP9^cko/CD4^ cells compared with wild-type counterparts (Fig. 6b). This suggests that dysregulated antigen-induced TCR upregulation in AKAP9^cko/CD4^ cells results in weaker signalling upon TCR re-stimulation and may thus account for the reduced re-stimulation observed *in vivo*.

To determine whether alterations in endosomal trafficking *per se* contribute to the hypo-responsive phenotype in AKAP9^cko/CD4^ cells, we co-cultured T cells with SEB-loaded splenocytes in the presence of the dynamin inhibitor dynasore, which inhibits clathrin-dependent coated-vesicle formation^50^ and thus endosomal trafficking. BrdU uptake was significantly reduced in vehicle-treated AKAP9^cko/CD4^ when compared with AKAP9^wt^ cells (Fig. 6c). Although dynasore treatment decreased cell viability *per se*, proliferation of dynasore-treated cells was equivalent in AKAP9^cko/CD4^ and AKAP9^wt^ cells (Fig. 6c), which suggests that AKAP9-dependent endosomal trafficking is necessary for optimal T cell activation.

To elucidate the cause of reduced TCR upregulation in AKAP9^cko/CD4^ T cells following stimulation with SEB-loaded DCs, we examined TCR trafficking following its internalization upon CD3ɛ crosslinking. The advantage of this approach is a timed, synchronized generation of TCR endosomes. Furthermore, it enables us to observe endosomal trafficking after treatment with relevant inhibitors. The fate of internalized TCR was analysed by co-localization with the early endosome marker TfR and the late endosomal marker LAMP-1 (ref. 51). Despite similar internalization of surface TCR following CD3ɛ crosslinking in both AKAP9^wt^ and AKAP9^cko/CD4^ cells (see Fig. 6e for details), significantly more TCR co-localized with TfR and less with LAMP-1 in AKAP9^cko/CD4^ versus AKAP9^wt^ cells (Fig. 6d and Supplementary Fig. 5, analysis via Pearson's R), suggesting an accumulation of TCR in early endosomes in the absence of AKAP9. Studies investigating IL-4 receptor recycling have shown early to late endosomal formation to be MT-dependent^51^. Depolymerization or stabilization of MTs in AKAP9^wt^ cells after CD3ɛ crosslinking, using nocodazole or taxol, respectively, resulted in an endosomal distribution resembling that in AKAP9^cko/CD4^ T cells (Fig. 6a) while having no further effect in AKAP9^cko/CD4^ cells. Thus, MTs, and potentially AKAP9 regulation of MTs, is required for endosomal trafficking of internalized TCRs.

To investigate contributions of AKAP9 in TCR recycling from early endosomes to the cell surface, we examined TCR re-surfacing following its downregulation via CD3ɛ-crosslinking^51^,^52^ in the presence of cycloheximide to rule out effects of new protein synthesis on TCR levels. Within 2 h after crosslinking, the surface pool of TCR significantly recovered in both AKAP9^wt^ and AKAP9^cko/CD4^ T cells. This largely relied on Golgi-dependent trafficking as treatment with monensin impaired re-surfacing in wild-type cells (Fig. 6e). Monensin treatment of AKAP9^cko/CD4^ cells almost completely prevented TCR reappearance at the surface (Fig. 6e), indicating that Golgi-independent recycling is dependent on AKAP9. The protein synthesis- and Golgi-independent trafficking of the TCR was blocked by the MT depolymerizing agent nocodazole in AKAP9^wt^ cells with no further reduction in AKAP9^cko/CD4^ cells, inferring that AKAP9-dependent receptor recycling is MT dependent. To confirm these results with an independent approach, we incubated cells with biotin-conjugated anti-TCR and stained the remaining or re-surfaced TCR with streptavidin-phycoerythrin (PE) at different time points^51^. Again, AKAP9^cko/CD4^ cells had decreased re-expression of internalized TCR at the surface after 2 h and TCR surface re-expression in AKAP9^wt^ could be inhibited by nocodazole to levels observed in AKAP9^cko/CD4^ cells (Fig. 6f). Together these data indicate that MT-dependent recycling of endocytosed TCR to the surface is impaired in AKAP9^cko/CD4^ T cells and likely contributes to the observed accumulation of TCRs in early endosomes in these cells (Fig. 6d).

T-cell activation has been shown to require MT growth mediated by the MT plus end-binding protein 1 (EB1), which directly binds the TCR and facilitates TCR sorting and trafficking of the signalosome at the immunological synapse (IS)^53^. To examine the contribution of AKAP9 to association of the TCR with the MT network, we performed a MT co-sedimentation assay. TCRβ was present in the polymerized MT pellet of both AKAP9^cwt^ and AKAP9^cko/CD4^ samples (Fig. 6g). Similarly, comparable levels of EB1 were associated with immunoprecipitated TCRβ (Fig. 6h). These data indicate that AKAP9 is not essential for MT integrity and association with the TCR complex.

Taken together, these data suggest that recycling of early from early endosomes back to the cell surface upon TCR engagement requires AKAP9. TCR trafficking through endosomal compartments require MTs, the pharmacological disruption of which does not further impair recycling in AKAP9^cko/CD4^ T cells, suggesting that AKAP9 modulation of MT dynamics may be a prerequisite for efficient TCR recycling and signal propagation following TCR engagement.

## Discussion

We demonstrate an important role for the A-kinase anchoring protein AKAP9 in re-stimulation and subsequent retention of T cells at sites of inflammation using mice with deletion of AKAP9 in T cells. The idea that T cell effector functions are regulated by tissue retention is just beginning to emerge^9^. We show that impaired retention in both a model of glomerulonephritis and EAE associates with a reduction in disease progression. Adoptive transfer approaches in these models and in an acute model of renal ischaemia reperfusion demonstrates no effect of AKAP9 deficiency on T cell recruitment into tissues. Instead, AKAP9^cko/CD4^ T cells exhibit enhanced exit to lymphatics and accumulation in dLNs compared with their wild-type counterparts, which correlates with an overall reduction in TCR signal strength in response to antigen. *In vitro*, AKAP9^cko/CD4^ T_H_1 effector T cells proliferate less only following antigen presentation by non-classical APCs and have a defect in TCR endosomal trafficking to the surface. Thus, successful re-stimulation in non-lymphoid tissue under suboptimal antigen-presenting conditions may require AKAP9-mediated endosomal recycling of endocytosed TCR, whereas this process may be dispensable for contacts with DCs during the primary T cell activation event in the lymph node. We posit that non-classical APCs may be more dependent on AKAP9-mediated TCR recycling for optimal T cell activation as they are markedly less efficient on a cell-for-cell basis compared with lymphoid DCs, which are superior in their ability to express co-stimulatory molecules and process antigen compared with other APCs^54^.

Upon antigen recognition, supramolecular clusters of TCRs, co-receptors and signalling molecules accumulate at the T cell–APC contact to form the IS^55^. Activation of LFA-1 at the IS is required for conjugate formation^23^. A defect in LFA-1 positioning and activation at the IS was described in human T cell lines overexpressing a C-terminal fragment of AKAP9 or in cells with partial silencing of AKAP9, albeit no changes in conjugate formation were reported^19^. We show that in primary murine T cells, AKAP9 was not required for conjugate formation. Accordingly, immune cell populations were normal in mice lacking AKAP9 exclusively in T cells. AKAP9 in the cis-Golgi controls the assembly of a subset of MTs originating from this MT organizing centre in non-immune cells^15^ and Golgi nucleated MTs are crucial for the intracellular organization and polarized recycling of TCR complexes at the IS^56^. In particular, interaction of TCR with the MT plus-end binding protein 1 (EB1) mediates the polarized trafficking of TCRζ and linker for T-cell activation (LAT)-carrying vesicles to the IS^53^. As TCR interaction with EB1 was not AKAP9-dependent and plate-bound T cell stimulation did not reveal any defects in proliferation and proximal signalling pathways, our data argue against a role for AKAP9 in regulated trafficking of signalling vesicles to the IS, a prerequisite for effective activation of proximal signalling cascades^53^,^55^,^57^. Centrosomal AKAP9 has been implicated in cell cycle progression in some^58^ but not other cases^16^. However, the viability of AKAP9-deficient mice^21^ and the normal T cell counts in AKAP9 mice shown here suggest that AKAP9 in general is largely dispensable for cell proliferation.

Our data indicate that AKAP9 contributes to MT-dependent trafficking of endocytosed TCR complexes, both in their recycling back to the surface, attributed previously to Golgi-dependent MTs in the IS^56^, and in their transition to late endosomes. Internalization and recycling of TCRs during antigen presentation is well-accepted to play a role in IS formation^59^, however, the importance of signalling events associated with different endosomal compartments for specific downstream T cell activation events has not been established. The contribution of endosomes to signalling pathways may be cell type and receptor specific. For example, epidermal growth factor receptor (EGFR) requires early endosomes for efficient signalling^60^, whereas mammalian target of rapamycin complex 1 (mTORC1) requires the formation of late endosomes^61^. Our results infer that recycling endosomes may contribute significantly to signal integration during T cell activation, particularly in a setting with suboptimal co-stimulation as observed with non-classical APCs^41^. This infers that robust co-stimulation may overcome reduced TCR signal strength because of impaired recycling, consistent with an accepted relationship between co-stimulation and TCR signalling strength^62^. Recycled TCR from early endosomes influences IS formation and signalling by targeted, polarized recycling of endocytosed receptor to the site of antigen contact^59^. Interestingly, the authors of ref. 59 hypothesized that endocytosed TCR complexes may accumulate signalling molecules like CD45, Lck and LAT during recycling. An extension of this concept is that recycling may constitute a way of receptor ‘priming' as recycled complexes may return to the IS preassembled with key signalling components.

Recent studies have demonstrated that serial brief contacts of both CD4^+^ and CD8^+^ effector T cells with APCs are required to productively activate T cells in tissues (that is, translocate the transcription factor nuclear factor of activated T cells (NFAT) to the nucleus^7^,^8^), indicating that signal integration in between contacts is necessary to achieve activation. Although comparable studies for naïve T cells in the lymph node have not been reported, it is known that T cell contacts with DCs are less dynamic, and can last for hours^63^,^64^. Therefore, the mechanisms of activation at the site of inflammation may differ significantly from the primary activation of T cells in lymph nodes, an area that requires further study. Indeed, the cellular identity of APCs in the tissue in many cases remains to be identified. Relevant to our results, kidney DCs are less potent T cell activators compared with conventional DCs^42^ and APCs in the spinal cord have been described to be of phagocytic origin, a cell population that provides weaker co-stimulatory stimuli compared with DCs^65^. These differences may explain why AKAP9 deficiency has no effects on T cell differentiation and expansion in lymphoid tissue but has significant effects on suboptimal antigen-dependent activation, namely re-activation in inflamed, non-lymphoid tissue.

Mechanisms responsible for T cell retention are still largely unknown. The chemokine receptor CCR7 is essential for egress from the skin and lung^10^,^11^, albeit CCR7 expression on effector memory cells circulating through non-lymphoid tissue is low compared with naïve cells and central memory T cells^66^,^67^. Although T cell exit from peripheral tissue has been shown to be antigen-specific and CCR7-dependent^10^,^11^, to our knowledge, there is no evidence of CCR7 regulation at the site of inflammation. Our observations argue that another, yet to be identified molecular pathway also controls lymphocyte egress from the tissue downstream of TCR engagement and signalling.

In conclusion, our results indicate that AKAP9 is required for effective re-stimulation of effector CD4+ T cells in non-lymphoid tissue through regulation of endosomal trafficking of the TCR, a MT-dependent process. These results indicate that in addition to the described role of chemokine receptor/ligand interactions in regulating T cell egress^9^, AKAP9-dependent endosomal trafficking and signalling are essential for T cell re-activation under sub-optimal antigen-presenting conditions and the subsequent retention of T cells at sites of inflammation. Our data in crescentic glomerulonephritis and EAE indicate that impairments in these processes potentially have implications for disease pathogenesis.

## Methods

### Mice

All mice were housed under pathogen-free conditions. All animal experiments were approved by the Harvard Medical School Animal Care and Use Committee.

### Reagents and antibodies

All chemicals were obtained from Sigma-Aldrich and antibodies were purchased from BioLegend if not stated otherwise. Antibodies for p-ZAP70 (1:500), p-ERK (1:1,000) and t-ERK (1:2,000) were from Cell Signaling and anti-EB1 (1:500) from Absea.

### Generation of T cell-specific AKAP9 knock-out mouse

Mice with a floxed AKAP9 allele containing a lacZ and neomycin resistance cassette were generated from JM8.F6ES cells containing the AKAP9 transgene (The KOMP repository, University of California Davis and Children's Hospital Oakland Research Institute, California, USA) into C57Bl/6 Albino mice blastocysts. Chimeric animals were screened for germline transmission and offspring were bred to obtain homozygosity, bred with CAG-flp on a C57Bl/6J strain (PMID: 16651697; gift of Dr Shigeyoshi Itohara, RIKEN Institute, Japan) to excise the lacZ/neomycin cassette and then crossed with CD4-Cre recombinase mice (Jackson Laboratory) to obtain AKAP9 deficiency selectively in CD4^+^/CD8^+^ T cells^22^. Details are in Supplementary Fig. 1. CD4 genotyping was performed as per manufacturer's protocols (Jackson Laboratory), AKAP9 transgene PCR was done in two reactions. Primer pair 1 spanning the floxed site was 5′-CCAGTTGGGCTCCGCAAAGGA-3′ and 5′-AGTCTTCATCCAGATGCCCGACCT-3′. Primer pair 2 specific for the presence of the transgene was 5′-TGAAAATCCAGTTGGGCTCC-3′ and 5′-TCGTGGTATCGTTATGCGCC-3′. Wild-type controls for T cell conditional AKAP9-deficient mice (AKAP9^ckoCD4^) were either CD4-Cre animals or Cre-negative animals carrying the floxed allele. The control animals (with or without Cre) were phenotypically similar in all performed experiments.

### *In vitro* culture of T cells

CD4^+^ T cells were purified using a positive selection magnetic bead kit (Miltenyi) according to the manufacturer's protocol. The purified cells were then seeded on anti-CD3ɛ (Clone 145-2C11, 0.5 μg ml^−1^) precoated 24-well plates with the addition of 20 U ml^−1^ IL-2, 10 ng ml^−1^ IL-12 (R&D Systems), 0.5 μg ml^−1^ anti-IL-4 and 5 μg ml^−1^ anti-CD28 for T_H_1 differentiation and 20 U ml^−1^ IL-2, 5 ng ml^−1^ IL-4 (Peprotech), 0.5 μg ml^−1^ anti-IL-12 and 5 μg ml^−1^ anti-CD28 for Th_2_ differentiation. For co-culturing experiments, splenocytes were prepared by depletion of endogeneous CD4^+^ cells, RBC lysis and mitomycin C treatment (Tocris). DCs were derived from bone marrow using granulocyte–colony-stimulating factor (200 U ml^−1^) or, for proliferation assays following MOG immunization, purified from donor spleens using CD11c^+^ magnetic bead purification (Miltenyi). Antigen-presenting spleen cells or DCs were pulsed with 1 μg ml^−1^ staphylococcus enterotoxin B (SEB; List Biological Laboratories)^45^. For experiments using mixed lymphocyte populations^46^, splenocytes from BALB/c mice (Jackson Laboratory) were prepared from animals immunized with C57BL/6 splenocytes 7 days before isolation.

Cells were quantified by FACS as CD45^+^ TCRβ^+^ CD4^+^ events. In some experiments, cells were incubated with BrdU 72 h before analysis and additionally stained with anti-BrdU (BrdU Flow Kit, BD Biosciences) before FACS analysis or pulsed with 1 μCi of [3H]-thymidine for 16 h and harvested and quantified using an automated sample harvester (Perkin-Elmer).

### Conjugate formation

Conjugate formation was analysed as previously described^68^. Briefly, SEB-pulsed splenocytes or bmDCs were mixed with isolated T cells at given ratios (1:1 for bmDCs), centrifuged at 300*g* for 5 min and incubated for 30 min at 37 °C. Nonspecific conjugate formation was then disrupted by vigorous pipetting and conjugates were analysed by flow cytometry. Conjugates were identified via their forward scatter/side scatter (FSC/SSC) distribution and quantified as CD4^+^ TCR Vβ8.1/8.2^+^ events that were also CD11c positive.

For western blot analysis of signalling following conjugate formation, isolated T cells were sorted for TCR Vβ8.1/8.2, T_H_1 differentiated and mixed with DCs isolated from donor spleens (T cell:DC ratio 4:1). Cells were then centrifuged briefly (1 min, 1,400 r.p.m.) and incubated for the times indicated. The reaction was stopped by the addition of 6 × Laemmli buffer, incubation on ice for 10 min and boiling for 5 min. Uncut westernblots are supplied in Supplementary Fig. 6.

### Endosomal trafficking assay

Analysis of endosomal trafficking was done as previously described^51^. Briefly, following 2 h stimulation with plate-bound anti-CD3ɛ in the presence of cycloheximide (50 μg ml^−1^) and Bafilomycin A1 (100 nM, Wako Pure Chemical Industries), T cells were seeded on poly-L-lysine-coated coverslips in the presence of vehicle, nocodazole (20 μM), taxol (20 μM) or monensin (10 mM) for 30 min at 37 °C before fixation with 4% paraformaldehyde (PFA) in complete media. Fixed cells were then permeabilized with 0.5% Triton-X, blocked with 10% donkey serum and stained for LAMP1 (CD107a, 1:100), pan-TCR and transferrin receptor (TfR, 1:100, Abcam) followed by secondary antibodies conjugated to Alexa488, 568 and 647, respectively (1:500, Life Technologies) and counterstaining with 4,6-diamidino-2-phenylindole (DAPI). Mounted coverslips were then imaged by confocal microscopy (FV1000, Olympus) and analysed with FIJI distribution of NIH's ImageJ using the Coloc 2 plugin after renormalization with the adjust/threshold tool and background subtraction using the background subtraction feature of FIJI.

### T-cell receptor regulation

To examine T cell receptor upregulation, T cells were incubated in a 1:1 ratio with SEB-pulsed bmDCs overnight (12 h) and TCR Vβ8.1/8.2-positive cells were analysed for surface expression of the TCR β−chain or anti-CD3ɛ immediately after the overnight incubation or 4 h after transfer to a new plate and compared with expression before T cell–DC co-incubation. For re-surfacing experiments, T cells were activated via CD3 crosslinking by plating on anti-CD3ɛ-coated wells followed by incubation for 2 h at 37 °C in the presence of cycloheximide (50 μg ml^−1^). Cells were transferred to a fresh plate and incubated for the times indicated with or without the addition of nocodazole (20 μM) or monensin (10 mM) before T cell receptor surface expression was analysed using flow cytometry. In other experiments, cells were first stained with biotin-conjugated anti-TCR at 4 °C and then incubated for times indicated with or without monensin or nocodazole at 37 °C followed by staining with PE-Streptavidin at 4 °C to stain for surface TCR molecules.

### MT-binding assay

MT-binding assay was done as previously described^69^. Briefly, cleared T cell lysates were incubated for 20 min at 37 °C with or without 0.5 mM GTP in the presence of 20 μM Taxol and layered on a sucrose cushion. The pellet was washed once and analysed via western blot for α-tubulin and TCRβ (Santa Cruz).

### Air pouch model

The air pouch model was performed following the formation of the air pouch by two consecutive injections of air on day −6 and −3, and instillation of 0.5 ml containing 500 ng of recombinant murine TNFα or PBS alone plus 2.5 ml air on day 0. Mice were euthanized 24 h later, the air pouch was washed three times with 3 ml of PBS/5% FCS and recovered cells were quantified via FACS.

### T-cell homing assay

Naïve T cell homing was examined as reported previously^70^. Briefly, naïve T cells were labelled using CellTracker CMTMR and CMFDA probe (Life Technologies) according to the manufacturer's protocol, mixed in a 1:1 ratio and a total of 1 × 10^7^ cells was injected into the tail vein of a wild-type mouse. The animals were killed after 3 h and the competitive recruitment to peripheral (axillary and inguinal) lymph nodes and the spleen was analysed via flow cytometry. For half of the conducted experiments, celltracker colours were exchanged to exclude dye toxicity effects.

### Anti-GBM nephritis model

Anti-GBM nephritis was induced as described previously^29^. Briefly, mice were preimmunized subcutaneously in the right footpad with 0.05 mg rabbit IgG (Jackson ImmunoResearch Laboratories Inc.) in Freund incomplete adjuvant and nonviable desiccated Mycobacterium tuberculosis H37Ra (Difco). Three days later, mice were injected intravenously with 50 μl heat-inactivated, filter-sterilized nephrotoxic serum. Spot urine samples were collected on days 0 and 10 after injection of nephrotoxic serum. Sham animals were immunized but did not receive nephrotoxic serum. Kidneys from euthanized mice were harvested for histological analysis and flow cytometry. Urine albumin concentrations and creatinine levels in urine and serum were determined by ELISA (Bethyl Labs) and Creatinine Assay Kit (Cayman Chemical Company), respectively. Albuminuria was expressed as milligrams albumin per milligrams urinary creatinine to standardize urine albumin excretion for glomerular filtration rate and urinary concentration. Leukocyte infiltration was analysed using flow cytometry following digestion with Collagenase XI (125 U ml^−1^), Hyaluronidase I (60 U ml^−1^) and DNAase I (20 U ml^−1^) for 45 min and mincing through a 70-μm cell strainer. For evaluation of cytokine-producing cells, kidney lysates were incubated with PMA (20 ng ml^−1^)/ionomycin(1 μg ml^−1^) in the presence of Brefeldin A for 6 h before staining.

### Renal ischaemia reperfusion injury model

Briefly, mice were anaesthetized with intraperitoneal injections of ketamine and xylazine and placed on a heating pad to maintain body temperature. In animals undergoing renal ischaemia-reperfusion, both renal pedicles were clamped off for 32 min with haemostatic microclamps. Kidneys were inspected for immediate colour change, indicating successful clamping. After clamp removal, kidneys were checked for a change in colour within 3 min to ensure reperfusion. In animals subjected to sham operation, the surgical procedure was identical except that no clamps were applied. Incisions were closed in two layers and animals were allowed to recover. Three or twenty-four hours after reperfusion, the mice were euthanized and kidneys were harvested. For BrdU incorporation experiments, mice were injected i.p. with 200 mg of BrdU after reperfusion as well as 20 h after reperfusion and analysed 4 h after the last injection of BrdU.

In some experiments, *in vitro* differentiated, T_H_1 cells labelled with Celltracker CMTMR and CMFDA (Life Technologies) according to the manufacturer's protocol were adoptively transferred at a 1:1 ratio into mice induction of EAE, anti-GBM or ischaemia-reperfusion injury via tail vain injection or, in the case of ischaemia-reperfusion injury, 12 h after reperfusion via subcapsular renal injection. Animals were analysed at 3 h, and in the case of ischaemia-reperfusion injury, 24 h for recovery of transferred cells from the kidney, dLNs and peripheral lymph nodes or, in the case of subcapsular injection, 12 h after injection from draining and peripheral lymph node. Dye colours were exchanged for every other experiment to account for variations in dye toxicity. In some experiments, kidneys were injected with 5 μg SEB subcapsularly following reperfusion. Transferred T_H_1 cells labelled with CFSE (Life Technologies) were then injected and recovered cells were gated on TCR Vβ8.1/8.2 and analysed for expression of Nur77.

### EAE model

EAE was induced in female mice as previously described^39^. Briefly, mice were immunized s.c. with 100 μg of MOG (35–55) peptide emulsified in CFA (H37Ra; Difco Laboratories) and treated with 200 ng i.p. of pertussis toxin (List Biological Laboratories Inc.) on day 0 and day 2 post-immunization. Mice were observed daily up to 30 days and graded as follows: 0, no disease; 1, limp tail or isolated weakness; 2, partial hind limb paralysis; 3, total hind limb paralysis; 4, total hind limb and partial forelimb paralysis; 5 moribund or death. For histological studies, mice were killed on day 30 and spinal cords were fixed in Bouin's solution and stained with luxol blue. For analysis of cytokine profiles, isolated cells from lymph nodes, spleens or spinal cords were stimulated *in vitro* with 20 ng ml^−1^ PMA and 1 μg ml^−1^ ionomycin for 6 h in the presence of brefeldin A before staining.

### Kidney slices

Viable organ slices were prepared as described previously^4^. Briefly, mice were killed and perfused with ice-cold PBS/EDTA, kidneys were then removed and embedded in 5% low melting agarose and cut into 100 μm slices with a vibratome. Prepared slices were then incubated with 1 μg ml^−1^ SEB for 30 min at 37 °C before co-incubation with *in vitro* differentiated, labelled T_H_1 cells for 30 min at 37 °C after centrifugation (400*g* for 5 min). Slices were then washed with media to remove excess T cells and imaged by confocal microscopy. Touching cell–cell borders over 5 μm in the *xy* dimension were counted as interacting cells.

### T-cell adhesion assay

Static T cell adhesion was examined as described previously^70^. Briefly, CFSE-labelled T cells were seeded on a 96-well plate precoated with 2 μg ml^−1^ ICAM-1 (R&D Systems), ICAM-1 and 10 nM CXCL-12 (Peprotech), ICAM-1/0.2 mM Mn^2+^ or BSA at 2 × 10^5^ per well. Cells were incubated for 15 min at 37 °C to allow adhesion and were then submerged in prewarmed PBS and inverted to allow detachment by gravity for 20 min. Cell adhesion was quantified via ELISA reading of CFSE fluorescence.

### T-cell migration assay

Evaluation of T cell migration in a chemotactic gradient was done as outlined previously^70^. Briefly, 2.5 × 10^5^ T cells were placed in the upper well of a 3-μm pore size Transwell plate (Corning) with or without 50 nM CXCL12 in the lower chamber. Following incubation for 120 min at 37 °C, cells in the bottom chamber were quantified via FACS.

### T-cell transmigration assay

To evaluate T cell transmigration, T cells labelled with Celltracker CMTMR and CMFDA (Life Technologies) were perfused over confluent, TNFα-activated SEND-1 cells (provided by Dr M. Gimbrone, Brigham & Women's Hospital, Boston, Massachusetts) and given time to adhere. After 5 min, 2 dyne per cm^2^ shear was applied and lymphocyte movement was recorded over 10 min. Migration velocity was calculated as the displacement over time of migrating cells and transmigration frequency was calculated as the percentage of adherent cells that completely transmigrated through the endothelial cell layer at any point during the observation period. For every other experiment, cell dyes were exchanged to account for differences in dye toxicity.

### Statistics

Statistical analysis was performed with SPSS (version 22.0). Differences between the groups were evaluated by one-way analysis of variance, Student–Newman–Keuls test, *t*-test (data presented as mean ±s.e.m.) and Mann–Whitney *U*-test (data presented as median± interquartile range (IQR)) where appropriate. Normal distribution was assessed via Shapiro–Wilk test and consecutive Q–Q plotting. Data are presented as mean±s.e.m. or median±interquartile range, and *P*<0.05 was considered statistically significant. For *in vivo* experiments, the provided *n* is the number of animals used per experiment. For *in vitro* experiments, *n* describes the number of independent experiments, each done in technical triplicates.

## Additional information

**How to cite this article:** Herter, J. M. *et al.* AKAP9 regulates activation-induced retention of T lymphocytes at sites of inflammation. *Nat. Commun.* 6:10182 doi: 10.1038/ncomms10182 (2015).

## Supplementary Material

Supplementary Information Supplementary Figures 1-6 and Supplementary Methods
