# Supplementary material for: AKAP9 regulates activation-induced retention of T lymphocytes at sites of inflammation
Source: Nat Commun. 2015 Dec 18;6:10182. doi: 10.1038/ncomms10182 (PMC4703868; doi:10.1038/ncomms10182)
Supplement: Supplementary Information — Supplementary Figures 1-6 and Supplementary Methods [file ncomms10182-s1.pdf]

Supplementary Figure 1

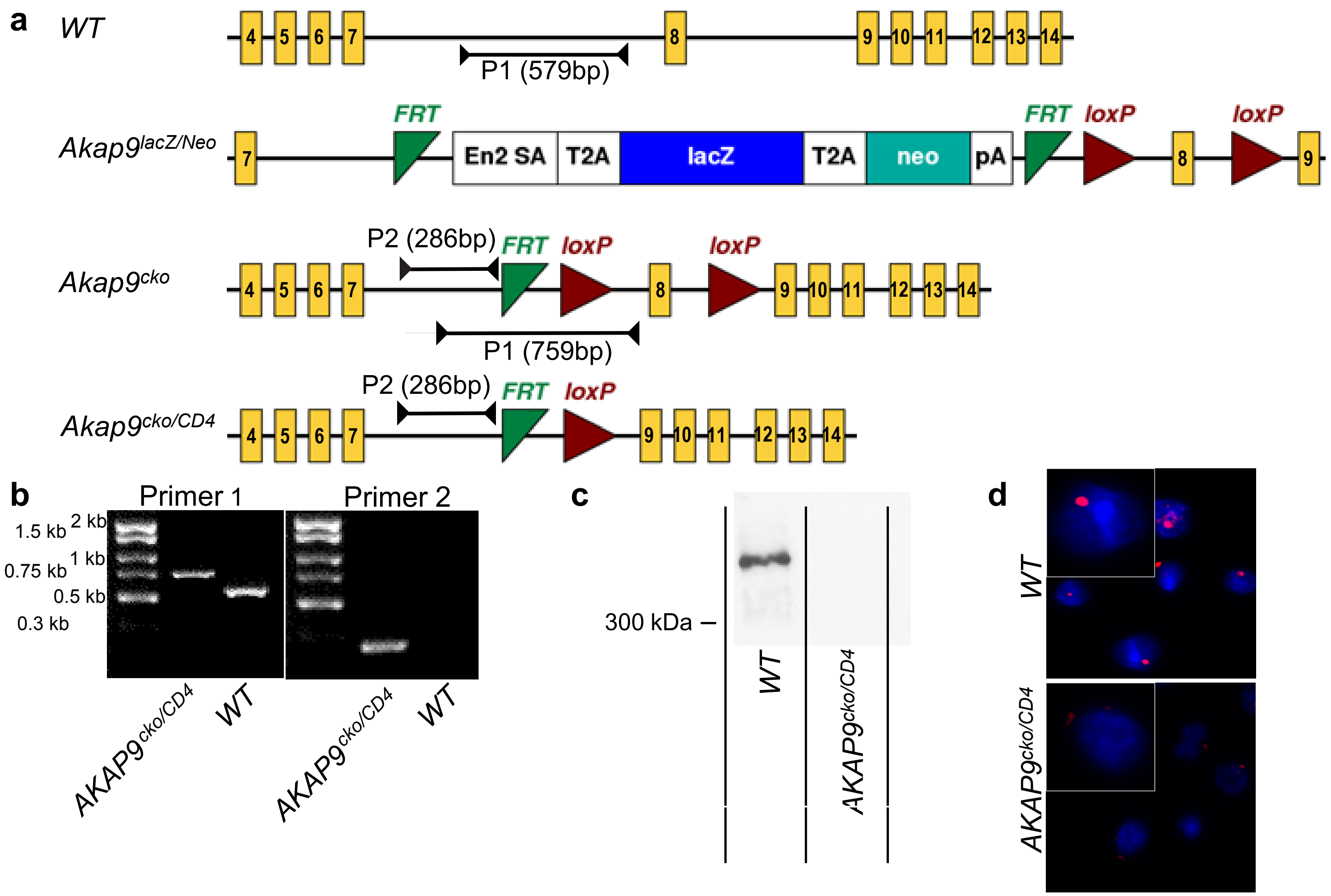

Supplementary Figure 1: Generation of T cell specific AKAP9 knock-out mouse. (a) Generation of AKAP9$^{cko/CD4}$ mice. WT refers to the wild-type allele. In mice expressing the targeted allele, AKAP9$^{lacZ/Neo}$ the lacZ/Neo cassette was excised using mice expressing flp recombinase to generate AKAP9^{cko} mice. AKAP9^{cko} mice were then bred with CD4-Cre mice to obtain AKAP9$^{cko/CD4}$ mice by excision of Exon 8. **(b)** Genotyping of DNA from tail biopsies of AKAP9$^{cko/CD4}$ mice and control mice. Primer pair 1 (PR1) binds after Exon 7 and before Exon 8 thereby spanning the loxP sites, yielding a 759bp fragment in mice with the mutant allele and a 579bp band for the WT allele. Primer pair 2 (PR2) binds after Exon 7 and within the loxP site yielding a PCR product of 286bp in mice with a floxed allele. **(c-d)** Verification of protein knock-down in purified T_H1 cells using an AKAP9 antibody that recognizes the N-terminus of AKAP9. Western blot analysis revealed that the expected 450kDa AKAP9 protein observed in wild-type samples was absent in AKAP9$^{cko/CD4}$ samples **(c)**. Immunofluorescence of T_H1 cells stained with AKAP9 antibody and counterstained with DAPI. Staining at the centrosome, observed in AKAP9^{wt} cells is largely absent in AKAP9$^{cko/CD4}$ cells **(d)**. # $p < 0.05$.

Supplementary Figure 2

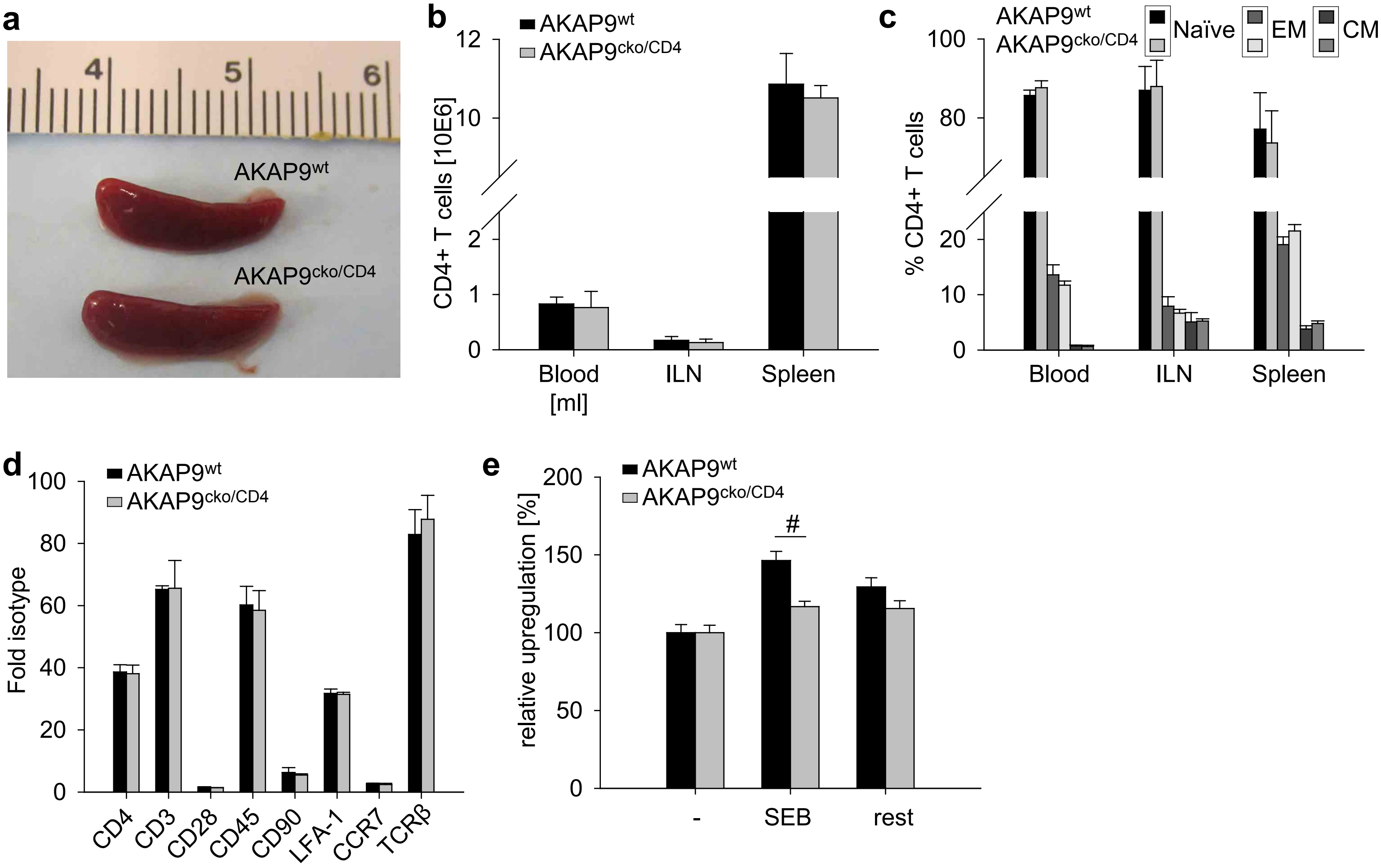

Supplementary Figure 2: CD4+ T cell counts and surface molecule expression. (a) Representative picture of a spleen from 8 week old male AKAP9$^{cko/CD4}$ and AKAP9^{wt} animals. **(b)** CD4+ T cell counts in mouse blood (per ml), the inguinal lymph node and the spleen as quantified via FACS ±SEM, n=4. **(c)** Mean percentage of naïve (CD62L^{high}CD44^{low}), effector memory (EM, CD62L^{low}CD44^{high}) and central memory (CM, CD62L^{high}CD44^{high}) CD4+ T cells of (b) ±SEM. **(d)** Expression of CD4, CD3, CD28, CD45, CD90, CD11a (LFA-1), CCR7 and TCRβ on splenic CD4+ T cells, presented as mean fold isotype staining ±SEM, n=3. **(e)** Upregulation of CD3ε after incubation with or without SEB loaded bmDCs as mean percent staining over baseline expression untreated cells and after 4h of rest ±SEM, n=4. # $p < 0.05$

Supplementary Figure 3

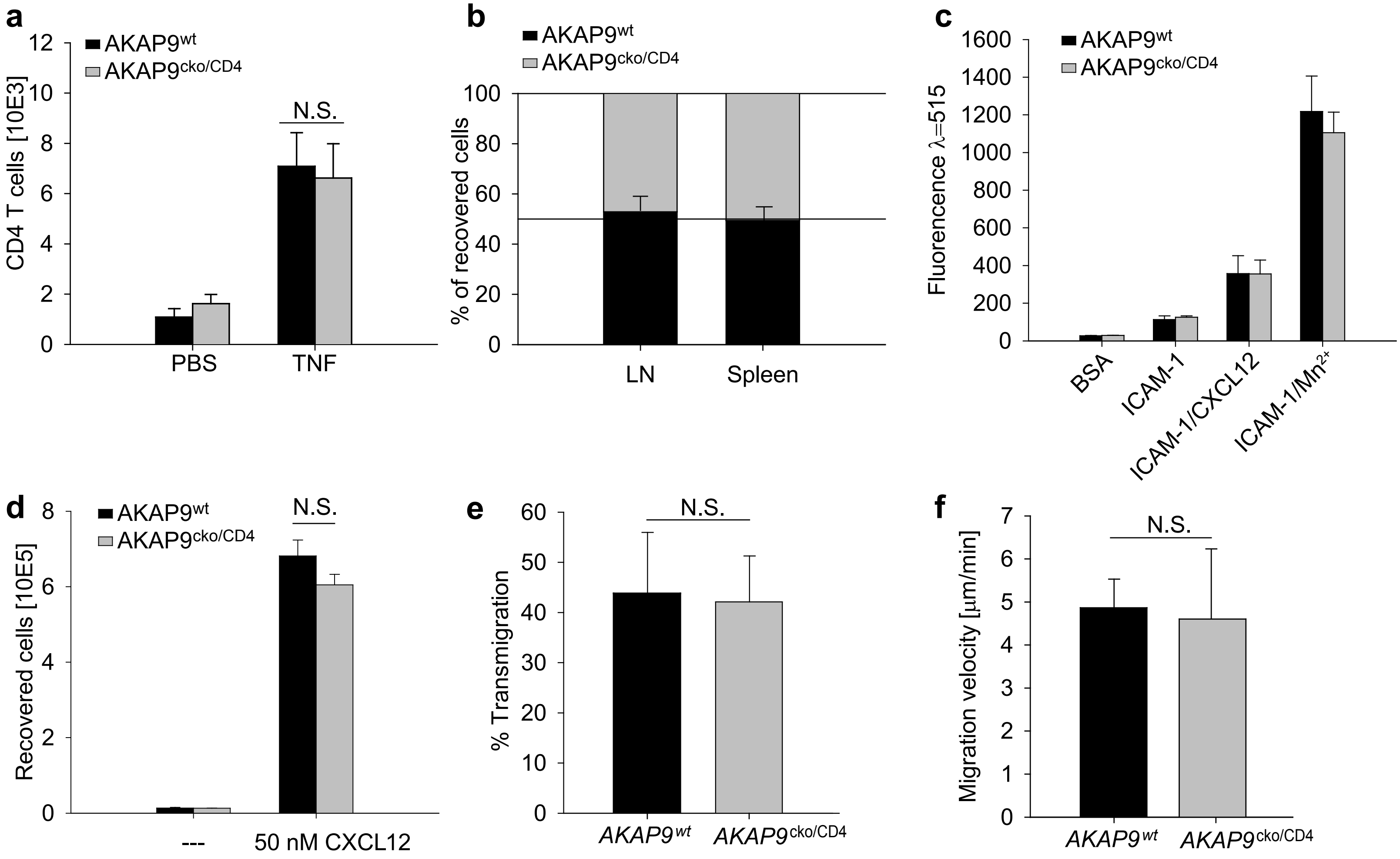

Supplementary Figure 3: AKAP9 deficient CD4+ T cells exhibit normal recruitment, homing, adhesion and migration. (a) T cell accumulation in the air pouch 24 hours after instillation of PBS or TNF in $AKAP9^{cko/CD4}$ and $AKAP9^{wt}$ mice, presented as mean number of cells ±SEM, n=5. **(b)** Naïve T cells of $AKAP9^{cko/CD4}$ and $AKAP9^{wt}$ mice, differentially labeled ex vivo, were co-transferred into wild-type recipient animals. Lymph nodes and spleens were analyzed for transferred cells 3 hours later via FACS, n=6. **(c)** T cell adhesion assay. CFSE loaded T cells were seeded on BSA or ICAM-1 coated 96 wells, with or without CXCL12 or Mn2+. Cells were then washed and fluorescence of bound cells was measured. Presented is mean fluorescence ±SEM, n=5. **(d)** Transwell migration assay. Purified, T cells were seeded on ICAM-1 coated transwells with or without CXCL12 in the bottom chamber and incubated for 90 min. Presented is mean number of recovered cells from the bottom well ±SEM, n=4. **(e)** Differentiated T_H1 CD4+ T cells from $AKAP9^{wt}$ and $AKAP9^{cko/CD4}$ mice were differentially labelled in vitro and co-perfused over TNF stimulated sEND1 endothelial cells and given 5 minutes to adhere. Cells were then recorded for 10 min and the number of transmigrated cells was determined. Presented is the mean percentage of all adherent cells that transmigrated during this time ±SEM, n=6. **(f)** Migration velocity of cells in (e) was quantified. Presented is the mean velocity of cells that adhered and moved during the observation time ±SEM.

Supplementary Figure 4

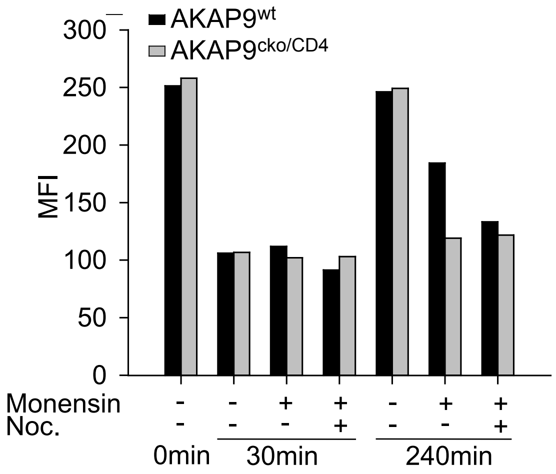

Supplementary Figure 4: Representative experiment of data depicted in Figure 6e. Graphed is the absolute MFI of TCR staining following CD3ε crosslinking.

Supplementary Figure 5

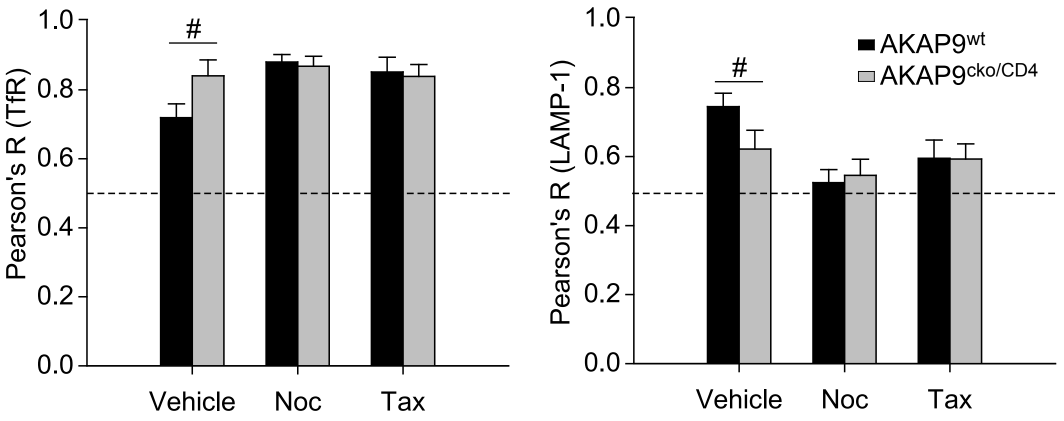

Supplementary Figure 5: Co-localization of TCR with TfR and LAMP-1. Computerized analysis of cells in Figure 6d using the Fiji Coloc2 plugin to assess colocalization. T cells were activated using plate bound anti-CD3ε/TCR and analyzed 30 min after replating. Quantification of co-localization of TCR with TfR (left panel) and LAMP-1 (right panel) after re-normalization to focus on endosomes. Data is presented as mean Pearson's R above threshold±SEM. Biologically meaningful co-localization are values >0.5 (dashed line). n=3, >50 cells/n.

Supplementary Figure 6

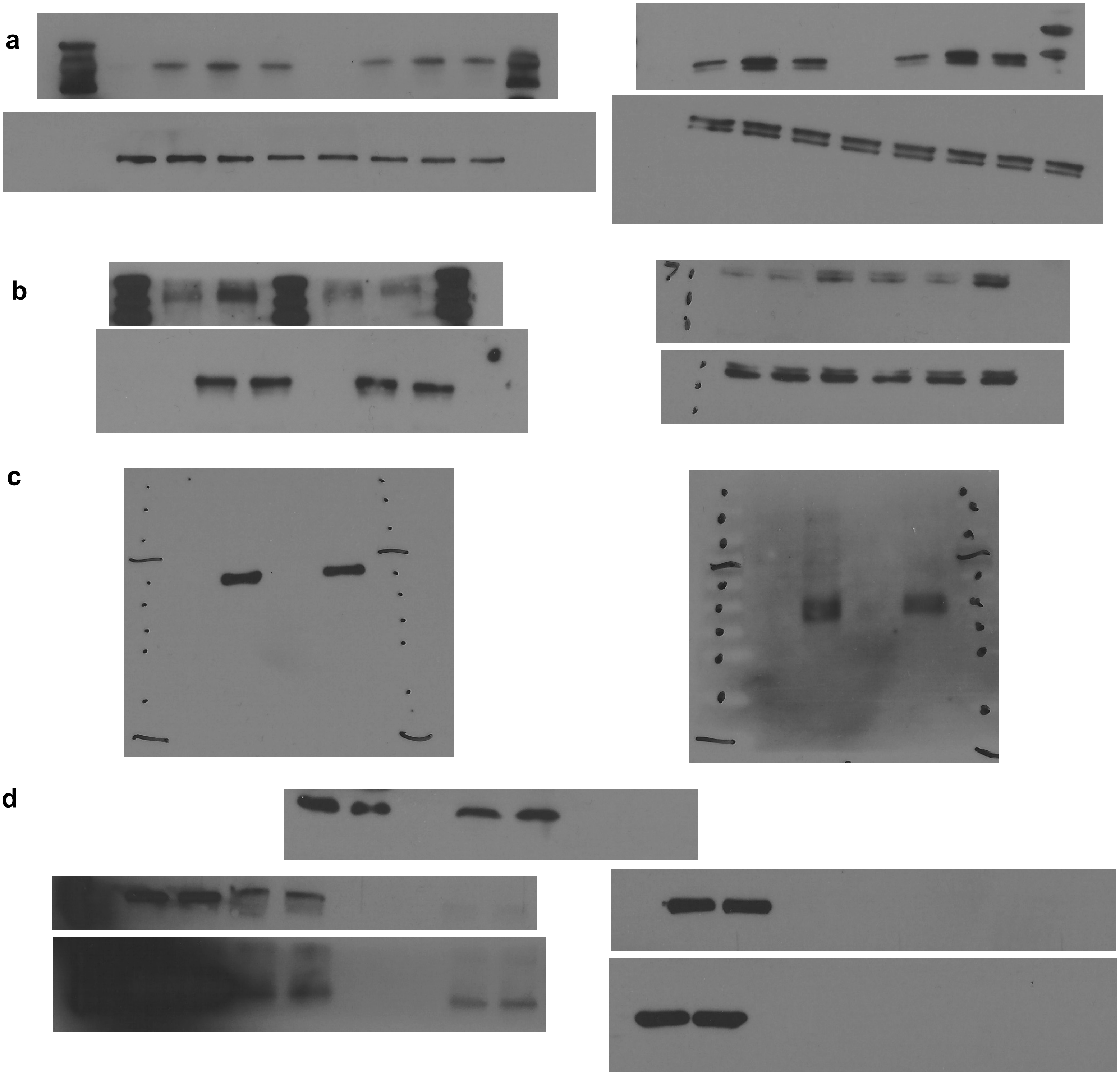

Supplementary Figure 6: Western blots. (a) Blots corresponding to Figure 5h. Left blots: upper panel is phosopho-ZAP70, lower panel is total ZAP70, developed was a membrane strip cut at the 55kDa and at the 100kDa band. Right blots: upper panel is phospho-ERK, lower panel is total ERK, developed was a membrane strip cut at the 55kDa band and at the 35kDa band. **(b)** Blots corresponding to Figure 6b. Left blots: upper panel is phosopho-ZAP70, lower panel is total ZAP70, developed was a membrane strip cut at the 55kDa and at the 100kDa band. Bottom blots: upper panel is phospho-ERK, lower panel is total ERK, developed was a membrane strip cut below the 55kDa band. **(c)** Blots corresponding to Figure 6g. Left is Tubulin, line annotations are 70kDa and 10kDa, dot below the 70kDa line is 55kDa. Right is TCRβ, band is between the 35 and 40kDa band. **(d)** Blots corresponding to Figure 6h. Top blot is CD3 loading control and IP, developed from a membrane strip cut below the 25kDa band as used to control for successful IP before blotting for ZAP70 and EB1. Lower blots left: exposures used for ZAP70 (upper) and EB1 (lower). Lanes 1 and 2 are loading controls, 2 and 3 are IPs, 4 and 5 are IgG control and 6 and 7 are IP but half of what was loaded in lane 4 and 5. Lower blots rights: loading control blots for ZAP70 (upper) and EB1 (lower). ZAP70 was developed from a membrane strip cut at the 55kDa and at the 100kDa, EB1 was developed from a membrane strip cut at the 55kDa band and at the 25kDa band.

Supplementary Methods

Generation of T cell specific AKAP9 knock-out mouse

Mice with conditional deletion of AKAP9 in CD4+ and CD8+ T cells (AKAP9$^{cko/CD4}$) were generated on a C57Bl/6 strain (Supplementary Figure 1a). A genotyping primer pair spanning the recombination sites yielded the expected 759bp fragment in mice with the mutant allele and a 579bp band in WT mice tail biopsies as expected (Supplementary Figure 1b left panel). Primers requiring the presence of recombination sites for binding yielded a 286bp fragment in mice with the mutant allele only (Supplementary Figure 1B right panel). Knock out was confirmed in CD4+ T cell lysates by western blot, which revealed the expected 450kDa AKAP9 protein in wild-type samples that was absent in AKAP9$^{cko/CD4}$ samples (Supplementary Figure 1c). Immunofluorescence of T cells using an N-terminal antibody for AKAP9 (Novus Biologicals, Cambridge, MA, USA) revealed a dense paranuclear signal in AKAP9^{wt} that was absent in AKAP9$^{cko/CD4}$ mice, consistent with a centrosomal localization. Residual diffuse staining was observed in the cytoplasm in both samples (Supplementary Figure 1d) that may reflect non-specific staining.

CD4 counts and surface molecule expression.

We found no differences in spleen size (Supplementary Figure 2a), total CD4+ T cell counts in the blood, inguinal lymph node and spleen (Supplementary Figure 2b), and distribution of L-selectin and CD44 on splenic CD4+ T cells (Supplementary Figure 2c). These data suggest that maturation and expansion of CD4+ T cells is not dependent on AKAP9. Furthermore, surface levels of T cell receptor molecules, co-stimulatory molecules and CCR7 were similar in *AKAP9*$^{cko/CD4}$ and *AKAP9*^{wt} mice (Supplementary Figure 2d).

AKAP9 deficient CD4+ T cells exhibit normal recruitment, homing, adhesion and migration.

To investigate T-cell accumulation at the site of inflammation, we examined recruitment to the air pouch 24 hours after installation of PBS or TNF in AKAP9$^{cko/CD4}$ and AKAP9^{wt} mice. Both groups showed similar recruitment to their pouch (Supplementary Figure 3a). To examine homing to lymph nodes, naïve T-cells of AKAP9$^{cko/CD4}$ and AKAP9^{wt} mice were differentially labeled ex vivo and co-transferred into wild-type recipient animals. Lymph nodes and spleens were analyzed for transferred cells 3 hours later via FACS. Both LFA-1 dependent homing to the lymph node as well as LFA-1 independent homing to the spleen were similar (Supplementary Figure 3b). To further exclude other contributions of AKAP9 to LFA-1 mediated T cell trafficking, we performed *in vitro* experiments. To examine adhesion, we performed a static adhesion assay on plate-bound ICAM-1. Following stimulation with CXCL12 or Mn^{2+}, we observed similar adhesion to ICAM-1 (Supplementary Figure 3c), indicating that both chemokine triggered activation of LFA-1 and binding of LFA-1 to ICAM-1 do not require AKAP9. We obtained similar results on the isolated ligands VCAM-1, E-selectin and P-selectin under flow (data not shown). Next, we investigated the ability of AKAP9 deficient CD4+ effector cells to migrate in response to a chemokine gradient. Using ICAM-1 coated transwell inserts, we observed similar migration of AKAP9 deficient cells and wild-type controls in response to CXCL12 (Supplementary Figure 3d), indicating intact polarization in response to the chemokine gradient and effective migration through the transwell. We then tested the ability of differentiated T cells to adhere to, migrate on and transmigrate through TNF stimulated endothelial cells. Again, we observed similar adhesion (data not shown), comparable transmigration rates (Supplementary Figure 3e) and similar migratory speed of cells on the endothelial layer (Supplementary Figure 3f). These data indicate that adhesion and migration both on the isolated but also on stimulated endothelial cells is not affected by AKAP9.

Co-localization of TCR with TfR and LAMP-1

To assess co-localization of TfR and LAMP-1 with the T cell receptor in a standardized manner, we used pixel-by-pixel comparison and determined the pearson’s R in a computerized manner of cells in Figure 5d using the Fiji Coloc2 plugin. We observed increased co-localization of the TCR with LAMP-1 in

AKAP9^{wt}, and increased co-localization with TfR in AKAP9$^{CD4/ko}$ cells (Supplementary Figure 5). It is noteworthy that biological meaningful co-localization is indicated by values >0.5. As both endosomal compartments are expected co-localize with the TCR, we expected values >0.5 in all groups.
